# VALTOCO^®^ (Diazepam Nasal Spray) for the Acute Treatment of Intermittent Stereotypic Episodes of Frequent Seizure Activity

**DOI:** 10.3390/neurolint13010007

**Published:** 2021-02-18

**Authors:** Elyse M. Cornett, Sam N. Amarasinghe, Alexis Angelette, Tunde Abubakar, Adam M. Kaye, Alan David Kaye, Elisa E. Neuchat, Ivan Urits, Omar Viswanath

**Affiliations:** 1 Department of Anesthesiology, LSU Health Shreveport, Shreveport, LA 71103, USA; samar1@lsuhsc.edu (S.N.A.); alexislangelette@gmail.com (A.A.); tabuba@lsuhsc.edu (T.A.); akaye@lsuhsc.edu (A.D.K.); ivanurits@gmail.com (I.U.); viswanoy@gmail.com (O.V.); 2 Department of Pharmacy Practice, Thomas J. Long School of Pharmacy, University of the Pacific, Stockton, CA 95211, USA; akaye@pacific.edu; 3 LSU School of Medicine, LSUHSC New Orleans, New Orleans, LA 70112, USA; 4 School of Medicine, Florida International University, Miami, FL 33199, USA; elisaneuchat@gmail.com; 5 Department of Anesthesia, Beth Israel Deaconess Medical Center, Critical Care, and Pain Medicine, Boston, MA 02215, USA; 6 Valley Anesthesiology and Pain Consultants–Envision Physician Services, Phoenix, AZ 85004, USA; 7 Department of Anesthesiology, University of Arizona College of Medicine-Phoenix, Phoenix, AZ 85724, USA; 8 Department of Anesthesiology, Creighton University School of Medicine, Omaha, NE 68124, USA

**Keywords:** valtoco, diazepam, seizure, GABA, bezodiazepine

## Abstract

Valtoco^®^ is a new FDA-approved nasal spray version of diazepam indicated for the treatment of acute, intermittent, and stereotypic episodes of frequent seizure activity in epilepsy patients six years of age and older. Although IV and rectal diazepam are already used to treat seizure clusters, Valtoco^®^ has less variability in plasma concentration compared to rectal diazepam. Furthermore, the intranasal administration of Valtoco^®^ is more convenient and less invasive than rectal or IV diazepam, making it ideal for self-administration outside of a hospital setting. Multiple clinical trials have taken place comparing Valtoco^®^ to the oral, rectal, and IV forms of diazepam. Aside from mild nasal irritation and lacrimation, Valtoco^®^ was found to have no increased safety risk in comparison to traditional forms of diazepam. This review of Valtoco^®^ will include a history of diazepam prescribing and withdrawal treatment, Valtoco^®^ drug information, its mechanism of action, pharmacokinetics and pharmacodynamics, and a comprehensive review of clinical studies.

## 1. Introduction

A seizure is a sudden and uncontrolled electrical disturbance in the brain that can cause changes in movement, behavior, feelings, and consciousness [1]. Based on the International League Against Epilepsy (ILAE) classification of seizures, which was updated in 2017, seizures can be classified as focal, general, or unknown onset [1]. The difference between these types of seizures is determined by where they originate in the brain. Focal onset seizures can originate in one area, hemisphere, or group of cells in the brain. Focal seizures can be classified as aware or impaired awareness [2]. Focal onset aware seizures occur when a person is awake and aware during the seizure. Focal onset impaired awareness seizures occur when a person is confused or their awareness is impaired. Focal onset seizures can have motor and non-motor symptoms [2]. Motor symptoms can include jerking, limp or weak muscles, and tense or rigid muscles. Non-motor symptoms include changes in sensation, emotion, thought, cognition, gastrointestinal symptoms, or a complete lack of movement. General onset seizures affect both sides of the brain (or groups of cells on both sides of the brain) at the same time [2]. General onset seizures have motor and non-motor symptoms. The motor symptoms are similar to focal onset seizure motor symptoms. The non-motor symptoms include staring spells or brief twitches that may affect only one part of the body (e.g., the eyelid) [2]. Unknown onset seizures occur when the cause of a seizure is not known. Usually, this category can be excluded as information is gathered from the patient or family members to narrow down how and why the seizure occurred. Unknown onset seizures can have tonic-clonic (what is generally recognized as a seizure during which the person loses consciousness or has stiff muscles and jerky movements) or epileptic motor spasms. The non-motor seizures in this category include the absence of behavior or staring [2]. Seizure clusters are seizures that start and stop and occur in groups one after another. A cluster can also be considered as two or three seizures in 24 h with recovery between each seizure.

When a patient experiences two or more seizures that are unprovoked, a diagnosis of epilepsy can be given [3]. The antiepileptic drug prescribed for treatment depends on the classification of the seizure. Carbamazepine or lamotrigine are recommended first-line treatments for focal seizures, while sodium valproate is a recommended first-line treatment for generalized tonic-clonic seizures [4,5,6]. For seizure clusters and status epilepticus, the recommended first-line treatment is a benzodiazepine (BZD), such as diazepam or midazolam [7,8,9,10]. Diazepam and other BZDs are used because they bind to gamma-aminobutyric acid (GABA)-A receptors, which causes increased chloride influx and hyperpolarization of the neuron, resulting in decreased neuron excitability and antiepileptic activity [11,12]. This review discusses the original use of diazepam and the epidemiology, pathophysiology, risk factors, presentation, and treatment of diazepam withdrawal. The present manuscript also describes Valtoco^®^, the nasal spray form of diazepam, and its clinical use for the acute treatment of intermittent, stereotypic episodes of frequent seizure activity, in addition to its mechanism of action, pharmacokinetics, and pharmacodynamics. Lastly, clinical trials of Valtoco^®^ will be compared to determine its safety and efficacy.

## 2. Diazepam Withdrawal

### 2.1. Epidemiology

Benzodiapeines, such as diazepam, have been approved for the treatment of anxiety, acute alcohol withdrawal, skeletal muscle spasm, and epileptic disorders, such as SE. They are also used for “off-label” treatment of conditions like insomnia [13]. In the past, barbiturates were used to treat these conditions, but benzodiazepines have largely replaced them due to their greater safety, lower abuse potential, and CNS specificity. As more conditions (e.g., Dalmane^®^ or Halcion^®^ for insomnia) have been approved or accepted clinically for treatment by BZDs, the amount of BZD prescriptions have increased. From 1996 to 2013, the amount of people filling a BZD prescription increased from 8.1 million to 13.5 million, a 67% change [14]. Similarly, the percentage of adults filling a BZD prescription increased from 4.1%, with an annual change of 2.5% from 1996 to 2013 [14]. In addition to increased prescription rates, a 29% increase in emergency department visits due to nonmedical use of BZDs was reported in 2011, representing a 149% increase compared to 2004 [15]. Although specific data for BZD use disorder in the United States is unavailable, the lifetime prevalence of sedative use disorders is estimated to be 1.1% [16]. Benzodiazepines are DEA class IV related to safety, misuse, and abuse potential.

### 2.2. Pathophysiology

While patients taking BZDs do not have to be addicted to experience withdrawal symptoms, withdrawal is common after long-term use. It often takes months to taper off of BZDs. Although the exact mechanism is unknown, BZDs increase dopamine levels in the mesolimbic reward system. The ventral tegmental area (VTA), which is part of the mesolimbic reward system, contains GABA interneurons, dopamine neurons, and glutamate neurons [17]. BZDs bind to a specific pocket of GABA-A receptors located between the alpha and gamma subunits. Within the VTA, GABA interneurons with high numbers of GABA-A receptors that contain the alpha-1 subunit were found in mice [17]. The alpha-1 subunit has specifically been implicated in addictive behavior [18]. Once the BZD has bound to the GABA-A receptor, the release of GABA onto dopamine neurons is decreased. This results in disinhibition, since the inhibitory effect of GABA interneurons to dopamine neurons is decreased, leading to increased dopamine release [17].

Prolonged use of BZDs like diazepam result in conformational changes in the GABA-A receptor. Studies involving mice that were administered BZDs showed decreased mRNA levels of GABA-A subunits gamma-2 and alpha-1, while mRNA levels of subunit alpha-5 increased [19,20,21]. Allosteric uncoupling of the GABA-A subunits was also observed in mice that were administered BZDs [21,22,23]. These results suggest a mechanism of tolerance to BZDs, but do not explain the dependence after withdrawal from BZDs, since GABA-A subunit levels return to control levels within 72 h of discontinuation of BZDs in mice [21,24].

Benzodiazepines are also thought to alter synaptic plasticity via alpha-amino-3-hydroxy-5-methyl-4-isoxazole propionic acid (AMPA) receptor migration. BZD administration in mice has shown increased AMPA receptor migration from the interior of dopamine neurons to the surface [17]. When these AMPA receptors migrate to the surface of dopamine neurons in the VTA, they are more likely to be stimulated via glutamate, leading to increased dopamine levels [25]. In studies in which mice were administered BZDs and then observed at the cessation of BZDs, an increase in AMPA receptors at the surface of hippocampal CA1 neurons was noted [26,27,28]. This results in hippocampal hyperexcitability, and suggests that increased AMPA receptors at CA1 neurons may contribute to the anxiety symptoms experienced with BZD withdrawal [21,26], and are a potential mechanism of physical dependence [21,29].

### 2.3. Risk Factors

Multiple risk factors have been identified in regard to BZD dependence. One study that surveyed 599 BZD users showed that the main risk factors in decreasing order of significance are: being a member of a self-help group for medication dependence, younger age, longer time period of BZD use, higher dose of BZD, the interaction of higher BZD dose with a longer time period of BZD use, lower education level, non-native cultural origin, and outpatient treatment for alcohol and/or drug dependence [30]. Another study that interviewed 401 BZD users showed that patients with insomnia, concurrent antidepressant use, and alcohol dependence were at a higher risk of developing BZD dependence [31].

Another study that surveyed 43,093 adults representative of the United States population showed that BZD use disorder displayed psychiatric comorbidity with antisocial personality disorder, bipolar I disorder, panic disorder with agoraphobia, other prescription drug misuse, and other substance use disorders [16]. In two separate studies that followed and surveyed patients at methadone maintenance clinics, BZD misuse was found at significantly high rates [32,33]. Additionally, high rates of BZD misuse were found among injection drug users [34].

In an analysis of 48 cases of seizures thought to have been caused by BZD withdrawal, brain damage, alcohol addiction, and electroencephalogram abnormalities were found to be risk factors for BZD withdrawal seizures [35]. In an analysis of 20 reports, including studies and large case series of BZD withdrawal, BZDs with a short half-life, high doses of BZD, a long period of BZD use, and abrupt cessation of BZD use were associated with increased BZD withdrawal severity [36].

### 2.4. Presentation

BZD withdrawal can result in a range of symptomatic patterns. Rebound anxiety with insomnia within 1–4 days of BZD cessation is the most common symptom pattern of withdrawal. In most cases, this lasts for 2–3 days [37,38]. In more severe cases, patients can experience a combination of anxiety, insomnia, panic attacks, irritability, tremors, diaphoresis, difficulty concentrating, nausea, vomiting, weight change, headache, heart palpitations, and muscle aches. These symptoms can last for 2–14 days after BZD cessation [37]. In extreme cases, seizures and psychosis have been observed in patients after BZD cessation [39]. The severity and duration of withdrawal symptoms are related to how long the time period of BZD use was, if the BZD had a short or long half-life, and what tapering schedule was used [40,41]. Although most patients will experience symptoms that last no longer than 1–2 weeks, some may have symptoms for an extended time period [42].

## 3. Current Treatment of Diazepam Withdrawal

The use of diazepam for more than 3–4 weeks is likely associated with withdrawal symptoms if the drug is discontinued quickly. Physical symptoms of withdrawal include abnormal body sensations, aches/pains, delirium, muscle spasms, anxiety and panic attacks, depression, nausea, insomnia, and Grand Mal seizures [14]. The side effects of withdrawal are both physical and psychological. Patients have reported depersonalization, visual disturbances, depression, and paranoia. They have also felt gastrointestinal symptoms, difficulty walking, insomnia, and photophobia. There appears to be three categories of withdrawal symptoms associated with diazepam, depending on the dose administered. The first is a sedative hypnotic stage when dealing with high doses of diazepam. Second, if the patient is on a low dose stage, the symptoms are milder. Third, patients taking any dosage have a risk of symptom re-emergence, which includes anxiety that continued indefinitely. These symptoms presented without a previous history of psychiatric symptoms [43]. To avoid withdrawal, diazepam has to be managed with gradual dose reduction through tapering or through maintenance treatment. Staged dispensing is another effective option, and can be regulated through local pharmacies. Tapering the drug can be more challenging, because it is dependent on the dose, duration, age, and tolerance of the patient. There are many factors that are involved in the strategy of tapering the drug to avoid withdrawal. The physician must consider the patient’s current dose, how long they were taking the drug, whether it was used in monotherapy or multidrug therapy, and if there is any other substance abuse [14]. Interventions, substitutions, psychotherapies, and pharmacotherapies can aid in reducing withdrawal symptoms physically and psychologically. Physicians must be cautious of patients that are “doctor shopping” to obtain more prescriptions.

Anticonvulsants can aid in successful BZD withdrawal if the patient is not addicted to any other substances. Antidepressants and beta blockers have shown no benefit to aid with withdrawal [43]. Flumazenil, a GABA_A_ receptor antagonist, subcutaneous or intravenous over four days, has helped patients rapidly withdraw from BZD. Unfortunately, the common side effect of this drug is seizures, therefore, it is not used often. Non-BZD hypnotics, such as promethazine or chlormethiazole, have been indicated in cases of withdrawal symptoms that include severe insomnia. Psychotherapy was shown to have better results in decreasing withdrawal symptoms than dose reduction alone. Specifically, cognitive behavioral therapy was found helpful. Maintenance therapy is used when patients are also dependent on alcohol or drugs. These patients often are on a high dose of BZDs, and they should be monitored for potential “doctor shopping”. These patients are difficult to manage and follow for reliable data. Overall, it is difficult to treat diazepam withdrawal. Besides symptomatic treatment, the most stable treatment is to taper the usage of diazepam. This has to be carefully followed and modified by the physician handling the case due to severe and potentially fatal side effects [43].

## 4. VALTOCO^®^ (Diazepam Nasal Spray) Drug Info

Valtoco^®^ is a form of diazepam that is administered intranasally. According to the FDA, it is approved for “acute, intermittent, stereotypic episodes of frequent seizure activity (i.e., seizure clusters, acute repetitive seizures) that are distinct from a patient’s usual seizure pattern in patients with epilepsy six years of age and older.” Valtoco^®^ is given in 5 mg and 10 mg doses. It is administered with a single spray in one nostril and a second dose when required 4 h later. See Table 1.

The recommended dose of Valtoco® is 0.2–0.3 mg/kg. The maximum dosage is two sprays for a single episode. It should not be used more than once every five days. It is available in 5 mg, 7.5 mg, and 10 mg strengths. There are risks associated with taking this drug with opioids. Opioids may result in sedation, respiratory depression, coma, and death. According to the FDA: “Observational studies have demonstrated that concomitant use of opioid analgesics and BZDs increases the risk of drug-related mortality compared to use of opioids alone.” Therefore, if Valtoco® is used in adjunctive therapy with other opioids, it should be prescribed at the lowest dose for the shortest time possible, and patients should be monitored [44].

Valtoco^®^ may induce CNS depression. This drug must be used cautiously if patients are planning on engaging in activities that require mental alertness. It should also not be taken with alcohol or other CNS depressants due to potential respiratory suppression. Using Valtoco^®^ has a risk of suicidal thoughts or behaviors. Studies have shown that using antiepileptic drugs, such as Valtoco^®^, has twice the risk of suicidal thinking. These side effects were seen as early as one week into treatment, and can continue throughout the course of medication [44].

Valtoco® can also increase intraocular pressure in narrow angle glaucoma, and is therefore contraindicated. It can, however, be used in patients with open-angle glaucoma if the condition is controlled. The drug is also contraindicated in patients with known hypersensitivity to diazepam [44].

Valtoco® cannot be used in neonates because of potentially fatal “gasping syndrome” if the neonate is underweight. Gasping syndrome is characterized by central nervous system depression, metabolic acidosis, and gasping respirations. This is due to Valtoco® being a benzyl alcohol-preserving drug. There is not enough data to confirm or refute the safe use of Valtoco® during pregnancy, but the drug is excreted in breastmilk. Related to these side effects, patients should be carefully monitored when taking Valtoco® [44].

## 5. Mechanism of Action

Valtoco^®^ binds to BZD receptors located between the alpha and gamma subunits of GABA_A_ complexes. The GABA_A_ receptor consists of five protein subunits arranged in a ring around a central pore. The five protein subunits include two alpha subunits, two beta subunits, and one gamma subunit. BZDs are allosteric GABA_A_ receptor modulators, and therefore do not bind to the active site and are not true agonists. BZDs increase the frequency of the chloride ion channel opening, thereby increasing the inhibitory effect of GABA on neuronal excitability. Upon GABA_A_ receptor activation, chloride ions flow into the cell. This causes hyperpolarization of the cell and an overall negative charge. Because they are allosteric activators, they do not directly open the chloride channel. BZD effects are especially pronounced in the limbic system, thalamus, and hypothalamus. BZD receptor agonists work through GABA_A_ receptors to promote sedation by inhibiting brainstem monoaminergic arousal pathways. This is possible through the facilitation of VLPO inhibitory GABAergic projections to arousal centers, such as the anterior hypothalamus TMN, the posterolateral hypothalamic hypocretin neurons, and the brainstem arousal regions, ultimately causing sedation [45].

Studies have compared intranasal diazepam to oral and rectal gel diazepam. Compared to oral diazepam, Valtoco^®^ has a slower t_max_ (time to reach maximum plasma concentration). Intranasal administration has similar t_max_ to the rectal gel. Variability (as defined by the percent coefficient of variation of the geometric mean) in the peak plasma concentration was higher in Valtoco^®^ than oral diazepam. The diazepam rectal gel showed the greatest variability. No major nasal irritation was documented by subjects that participated in the trials; mild complications included minor epistaxis that resolved within 1 min. The significance of the NCBI’s trial was that “Diazepam nasal spray shows predicable pharmacokinetics and represents a potential novel therapeutic approach to control bouts of increased seizure activity (cluster seizures, acute repetitive seizures).” It was shown to be acceptably safe, with less variability than the rectal diazepam route, and showed no damage to nasal mucosa [46]. The types of epileptic condition had no significant effect on the pharmacokinetics of Valtoco^®^ [47]. Valtoco^®^ is specifically marketed for the treatment of cluster seizures. These types of seizures require more hospital visits, and have a greater negative impact on patient lives. The use of antiepileptics and BZDs as “rescue medications” in acute situations can help avoid status epilepticus and decrease hospital visits due to seizures. In the United States, rescue medications are underused, and therefore incur higher healthcare costs due to repeated emergency room visits. Prior to Valtoco^®^, rectal diazepam gel was the only FDA-approved rescue medication for seizure clusters. The intranasal administration of Valtoco^®^ is more desirable by patients and has less variability than the rectal gel [8]. Diazepam nasal spray safety was consistent with the profile of diazepam [47].

## 6. Diazepam Original Use

In 1963, diazepam was made available publicly for patient use. Even though its exact mechanism of action was unknown for 15 years, it was still widely prescribed for anxiety [13]. Diazepam is still currently prescribed for anxiety, but current guidelines of use are better defined compared to in the 1960′s. For general anxiety disorder, diazepam is only recommended for short-term treatment of up to four weeks, as an initial supplement to SSRI or SNRI therapy, or if a patient has severe and disabling anxiety symptoms that have not responded to SSRI, SNRI, or other anxiolytic classes of medication [48,49,50,51]. Diazepam can also be used for the acute treatment of panic disorder with or without agoraphobia [50].

As previously mentioned, diazepam has been indicated for the treatment of alcohol withdrawal symptoms, such as autonomic hyperactivity, irritability, combativeness, hallucinations, seizures, and delirium [52]. Diazepam has also been shown to reduce the likelihood of relapse after three months of alcohol cessation when given as a 30-day treatment [53]. Diazepam can also be used for the treatment of opioid withdrawal syndrome [54]. Oxycodone and hydrocodone tolerance can be reversed by an acute dose of diazepam [55].

Another indication for diazepam is muscle spasms, spasticity, or rigidity. Children with spastic cerebral palsy experience muscle spasms and hypertonia that severely limit their mobility. One study has shown that children with spastic cerebral palsy who were given diazepam versus a control displayed a significant decrease in hypertonia, improved passive range of movement, and increased spontaneous movement [56]. Multiple studies have shown that tetanus patients given diazepam alone versus a combination of conventional anticonvulsants had a better chance of survival [57]. Another study has shown that non-relaxing pelvic floor tension myalgia can be treated via vaginally administered diazepam [58]. Diazepam has also been shown to reduce muscle spasms in a study that analyzed 13 patients with stiff-person syndrome, a rare condition that is characterized by intermittent spasms and stiffness of the axial muscles [59].

Intermittent, stereotypic episodes of frequent seizure activity, such as seizure clusters that are different from a patient’s typical seizure pattern, are indicated for treatment by diazepam. An analysis of multiple studies showed that the use of oral, rectal, and IV diazepam helped absorb seizure clusters, avoid progression to status epilepticus, and reduce emergency room visits [8]. Status epilepticus is defined as an episode of more than 30 min of continuous seizure activity or two or more sequential seizures that don’t fully recover in between within 30 min [3]. An analysis of multiple studies showed that diazepam was effective in preventing or treating status epilepticus [12].

## 7. Pharmacokinetics and Pharmacodynamics

### 7.1. Pharmacodynamics

According to the FDA: “The effects of diazepam on the CNS are dependent on the dose administered, the route of administration, and the presence or absence of other medications.”

Valtoco^®^ is formulated with Intravail A3 (n-dodecyl beta-D-maltoside) and vitamin E to enhance solubility and absorption. Intranasal BZD formulations rely on glycols as co-solvents. Three concentrations of diazepam, 5, 7.5, and 10 mg in a 0.1 mL solution, facilitate weight-based doses [46]. BZDs act as positive allosteric modulators on the gamma amino butyric acid GABA_A_ receptor [60]. GABA is the most common inhibitory neurotransmitter in the central nervous system, especially in high concentrations in the cortex and limbic system. GABA_A_ receptors contain two alpha subunits, two beta subunits, and one gamma subunit. Each receptor complex has two GABA-binding sites and one BZD binding site. The BZD binding site is in a specific pocket in between the alpha and gamma subunits. Within the alpha subunit of isoforms 1, 2, 3, and 5 resides a histidine residue that possesses a high affinity for BZDs [61]. This high affinity binding induces a conformational change in the receptor. This conformational change causes the receptor’s chloride channel to hyperpolarize the cell, and accounts for GABA’s inhibitory effect throughout the central nervous system [60].

### 7.2. Pharmacokinetics

BZDs are administered in a variety of ways (intramuscular, intravenous, oral, sublingual, intranasal, or rectal gel forms). The volume of distribution is dependent on the characteristics of the drug. The characteristics that influence the distribution are the lipid solubility, binding to plasma proteins, and molecular size. Because elimination half-life is directly proportional to the volume of distribution and inversely proportional to its clearance, preexisting illnesses, such as renal and hepatic disease, drastically affect the elimination half-life.

BZDs are well-absorbed by the gastrointestinal tract after oral administration. If the drug is given intravenously, it is able to bypass the GI tract and go straight to the brain and central nervous system. In intramuscular administration of diazepam, the absorption is slower and erratic. In other intramuscular BZDs (e.g., Lorazepam or Midazolam), the absorption is fast and complete.

BZDs and their metabolites are highly protein bound. They prefer to cluster in lipid rich areas, such as the central nervous system and adipose tissue. This is important, because the more lipophilic the drug is, the higher the rate of absorption. This also results in a faster onset of clinical effects. Most BZDs are oxidatively metabolized by the cytochrome P450 enzymes (phase I), conjugated with glucuronide (phase II), and excreted almost entirely in the urine [60].

Valtoco® pharmacokinetics are similar to most BZDs, but there are a few differences due to the intranasal route of administration.

In a pharmacokinetic study in healthy adults, “the highest plasma concentrations after nasal administration were at 1.5 h. The estimated volume of distribution of diazepam at the steady state is 0.8 to 1.0 L/kg. The absolute bioavailability of Valtoco^®^ relative to intravenous diazepam was 97%. The mean elimination half-life of diazepam following administration of a 10 mg dose of Valtoco^®^ was found to be about 49.2 h. In a pharmacokinetic study in patients with epilepsy, pharmacokinetic parameters were similar between seizure versus non-seizure states” [47]. The drug binds to plasma proteins.

Metabolism and elimination is accomplished through CYP2C19 and CYP3A4 in the liver. They are responsible for the initial oxidative metabolism. Diazepam is extensively metabolized to one major active metabolite, desmethyldiazepam, and two minor active metabolites, 3-hydroxydiazepam (temazepam) and 3-hydroxy-N-diazepam (oxazepam), in plasma. The metabolism of diazepam is primarily hepatic, and involves demethylation and 3-hydroxylation followed by glucuronidation. No inhibition was demonstrated in the presence of inhibitors selective for CYP2A6, CYP2C9, CYP2D6, CYP2E1, or CYP1A2, indicating that these enzymes are not significantly involved in metabolism of diazepam [46].

## 8. Clinical Studies: Safety and Efficacy

The treatment of seizures with intranasal diazepam is generally safer than IV BZD treatment and rectal formulations of diazepam. Diazepam is well-tolerated in the treatment of epileptic seizures, and it is commonly administered via IV in a hospital setting [62]. However, the IV formulation cannot be used in most out of hospital settings, and therefore an easier formulation and administration of diazepam is warranted. Furthermore, the administration of rectal or oral diazepam to a person in an active seizure state poses many complications, such as choking, inability to assess the rectum, which may lead to limited bioavailability of the medication, or even death. Intranasal administration of diazepam has been proven safe in these situations [46].

A randomized crossover trial studied the bioavailability and safety of intranasal diazepam compared to oral and rectal diazepam [46]. The sample size of 48 healthy subjects was included in this phase one single-dose, three-treatment study that consisted of a screening period, baseline period, and open-label treatment period. The onset of absorption was very quick in both the nasal and rectal formulations, but the bioavailability of the nasal spray was only 60% of that of the oral diazepam. There’s less variability in the bioavailability of the nasal spray compared to the rectal gel, which makes it easier to predict its dosing. The bioavailability of the nasal spray was at a consistent 60% compared to the marked variability that was observed in the rectal gel diazepam in the study. The study recorded 131 mild treatment-emergent adverse events (TEAEs) in 42 subjects and four moderate TEAEs in four subjects; 100% of the subjects experienced at least one TEAE, but this frequency was less than that observed in treatments with rectal gel or oral formulations. Some of the treatment-emergent adverse events reported by the subjects included but were not limited to somnolence, headache, hypotension, hypertension, nausea, and hematuria. No subject was discontinued due to adverse events from the treatment. Nasal irritation was assessed on a 0–5 scale, with 0 = normal-appearing mucosal, 1 = inflamed mucosa, 2 = minor bleeding stopping within 1 min, 3 = minor bleeding that stopped with 1–5 min, 4 = substantial bleeding for 5–60 min that did not require medical intervention, and 5 = ulcerated lesions with bleeding requiring medical intervention. No subject had a nasal irritation that exceeded 2 (minor bleeding stops within 1 min) on the 0–5 scale. Sedation was gauged on a 0–5 scale with 0 = alert, not drowsy, and normal conversation; 1 = awake and talking, but somewhat drowsy; 2 = napping or sleeping but easily awakened; 3 = sleeping, awakened only with a loud voice or shaking; 4 = sleeping and very difficult to awaken; and 5 = sleeping and cannot awaken. Subjects with the nasal spray diazepam did not exceed sedation of 2 (napping or sleeping but easily awakened), while, with subjects using the rectal gel, the sedation score was as high as 4 (sleeping and very difficult to awaken) [46].

Another randomized phase one crossover study assessed the pharmacokinetics and tolerability of intranasal diazepam and compared it to diazepam rectal gel [63]. Twenty-four subjects were involved in the study, which involved administration of 5 mg or 20 mg of intranasal spray and 20 mg of rectal gel. The maximum plasma concentration of the 20 mg nasal spray and 20 mg rectal gel was 378 ng/mL and 328 ng/mL, achieved within 1 h and 1.5 h, respectively. The study indicated that the bioavailability of 20 mg of intranasal diazepam was comparable to the bioavailability of 20 mg of diazepam rectal gel, regardless of the presence of nasal leakages. Mild to moderate TEAE was observed at least once in all of the participants, but resolved without the need for additional treatments. The most common of the intranasal spray TEAEs reported was lacrimation, which mostly resolved within 45 min. Nasal leakage was observed in 65% of the subjects within 5–60 min after administration, but this did not lead to impaired absorption of the diazepam. Other TEAEs observed included but were not limited to sneezing, dysgeusia, hypertension, and rhinorrhea. Somnolence and dizziness were reported by all treatment groups, with occurrence increasing with an increased dose and being greater with rectal gel. Nasal redness was observed in 32% of the subjects treated with 5 mg of intranasal spray and 48% of subjects treated with 20 mg of intranasal spray, and symptoms were highest within 30–60 min after administration. Evaluation with the Columbia Suicide Severity Rating Scale (C-SSRS) did not indicate any treatment-emergent suicide ideation or behaviors in the subjects [63].

A randomized open-label, six-sequence, three-way crossover study examined the effects and efficacy of intranasal suspension, solution, and intravenous diazepam [64]. The maximum plasma concentration and time to maximum plasma concentration of the intranasal diazepam solution and suspension were identical, while the systemic availability of the intranasal solution was 97%, and the availability of the suspension was only 67%. Elimination of the intranasal suspension, solution, and intravenous diazepam was similar. It is important in diazepam administration to note the time needed to attain the desired drug concentration. The intranasal suspension and solution attained their peak at an average of 60 min and 70 min, respectively. This study showed that a peak concentration of >100 ng/mL was maintained 8 h after the intranasal solution dosing; 71% of the subjects experienced more than one TEAE, which were mild to moderate in severity and self-limiting. The most common TEAEs reported were epistaxis and somnolence in all administrations, but the somnolence was more prevalent with IV diazepam. Other TEAEs experienced by subjects included headache and nasal discomfort [64].

A 2013 study compared the bioavailability and efficacy of rectal and intranasal formulations of diazepam in a crossover study. The study indicated that the times to maximum plasma concentration of both the nasal spray and rectal gel were identical, with a median of 0.75 h. The mean absorption and elimination of diazepam were similar for all forms, but this varied greatly by individual subjects. The mean maximum plasma concentration was similar for the 10 mg rectal gel and 10 mg nasal spray, but it was higher in the 13.4 mg nasal spray subjects. The study reported maximum concentrations in the range of 150–190 ng/mL in the intranasal administrations, which fell short of the proposed minimum target concentration reported in the literature. Furthermore, reaching the minimum effective concentration of the drug in a patient is more important than the time to reach the maximum plasma concentration. The authors proposed that a second administration of the intranasal spray 5 to 10 min after the first dose may be indicated in some patients. There were no severe adverse effects from the treatments. Subjects reported mild discomfort that lasted up to 5 min after administration and increasing sedation, which had its maximum effects 2 h post-administration. The increasing sedation did not correlate with the maximum plasma concentration or the dose concentration. None of the subjects were observed to have a change in nasal irritation that was worse than the baseline. This is easier than administering a repeated rectal gel dose, and is expected to be safe due to a wide therapeutic and safety window of diazepam reported in the literature [65].

A 2009 study compared the efficacy of intranasal and parenteral diazepam and midazolam in a crossover study. The study reported higher maximum plasma concentration for intranasal diazepam compared to intranasal midazolam, and increased time to reach maximum concentration for diazepam. Since diazepam has a longer half-life compared to midazolam, it has greater advantages in its use for the treatment of seizure activities. Subjects in both formulations reported considerable pain with the nasal administration of both drugs. Only four subjects were recruited for the study, with one subject dropping out due to non-treatment related reasons [66].

Sperling et al., 2014 reported on the dosing, efficacy, and tolerability of diazepam nasal spray in a study of 31 subjects who were known to have various seizure disorders; 87% of the patients had treatment-resistant epilepsy. Subjects’ antiepileptic drugs were altered to included reduction and complete discontinuation, and one subject’s was increased prior to their involvement in the study. Diazepam was at a mean concentration of 158 ± 57.2 ng/mL 15 min after administration, and reduced to a mean of 77 ng/mL 12 h after administration. The maximum plasma concentration was 208 ± 90.3 ng/mL after a time of 1 h. The maximum plasma concentration was similar when dosing during the ictal period within 5 min after the ictal period or greater than 5 min postictal period; 65% of the subjects did not experience new seizures during observation, while the remaining 35% of patients experienced at least one more seizure, with the average time of 4.8 h after administration, and 45% of patients with a second seizure had a lesser, simple, or complex partial seizure after intranasal diazepam administration. The study indicates that intranasal diazepam is safe to be administered with any type of seizure or at any point within the course of a seizure. There was no significant change in the patients’ breathing patterns after administration of diazepam; 90% of the patients had at least one adverse reaction. Headache was the most common, followed by dysgeusia, lacrimation, nausea, rhinorrhea, somnolence, and other mild symptoms. The majority of the adverse events were mild to moderate, with a single case of severe headache not related to treatment. Nasal irritation and inflammation from drug administration resolved within one day. There were no severe sedation effects from the drug, with most of the participants alert, while others were either drowsy or easily aroused from sleep. There was no suicidal ideation reported after evaluation with the C-SSRS [67].

A longitudinal, retrospective cohort study examined the difference in the cessation of status epileptics in stroke patients who were given either intranasal or intravenous diazepam over a five-year period at the University of Tokyo Hospital, Japan. Nineteen patients who fit the inclusion criteria of seizure activities for greater than 30 min without prior midazolam administration were included in the study. Nine patients received intranasal diazepam, while 10 patients received intravenous diazepam. The study reported that the intranasal diazepam delivery was nine times faster than intravenous delivery, which may account for the statistically significant cessation of seizure activities that occurred 3 min after arrival with intranasal administration, compared to 9 min after arrival with intravenous administration. Although the cessation of seizure activities occurred fast at 0.5 min with intravenous administration compared to 3 min with intranasal administration, this difference was not statistically significant. There were no reports of deaths or respiratory or hemodynamic compromise within 72 h of administration. Researchers proposed that the large nasal mucosa, which is highly vascularized and close to the brain, might explain the high rate of effectiveness of the intranasal diazepam [68].

Fifty-seven participants were involved in a study exploring the effects and safety of intranasal diazepam during seizure (ictal/peri-ictal) or non-seizure (interictal) conditions. Both adults and pediatric patients of an age range from 6 to 65 years with a previous clinical diagnosis of epilepsy were enrolled in this study. Subjects received either 10 mg, 15 mg, or 20 mg doses of intranasal diazepam. The time to maximum plasma concentration of the drug was reached approximately 2 h after dosing. The maximum plasma concentration was comparable for the ictal/peri-ictal and the interictal groups; 29.8% of the subject reported a treatment-emergent adverse event (TEAE), but none of these were severe or led to discontinuation of the subject in the study. The TEAEs reported included but were not limited to dysgeusia, seizure, nasal redness, and nasal discomfort. None of the subjects had a significant change in their electrocardiograms from baseline or evidence of respiratory depression. There were no reports of somnolence or suicidal ideation, although there were small increases in sedation more common with the ictal/peri-ictal administration, but this varied with individual subjects. The sedation was transient and was not dose-dependent. This study highlighted the efficacy and safety of intranasal diazepam in children and adolescents, who have been rarely included in many other studies for this drug [47]. See Table 2.

## 9. Conclusions

Diazepam has been widely used and prescribed since its release in 1963. It has helped treat anxiety, muscle spasms, alcohol withdrawal, and seizures for millions of people around the world. Despite diazepam’s ability to help alleviate patients’ suffering, its addictive properties have led to misuse and potentially severe withdrawal symptoms, like anxiety, insomnia, psychosis, and seizures [13]. Since treating diazepam withdrawal aside from symptom management has proven difficult, physicians have begun to question when it is absolutely necessary to prescribe BZDs like diazepam [43]. This has led to more strict guidelines for when to prescribe diazepam, especially in regard to treating anxiety [50]. Valtoco^®^, a new FDA-approved nasal spray version of diazepam, has been indicated for the treatment of acute, intermittent, and stereotypic episodes of frequent seizure activity in epilepsy patients six years of age and older [69]. Although IV and rectal diazepam are already used to treat seizure clusters, Valtoco^®^ has less variability in plasma concentration compared to rectal diazepam [46]. Additionally, the administration of Valtoco^®^ intranasally is more convenient and less invasive than rectal or IV diazepam, especially when a patient is actively seizing or not in a hospital setting [47]. Multiple clinical trials have taken place comparing Valtoco^®^ to the oral, rectal, and IV forms of diazepam. Aside from mild nasal irritation and lacrimation, Valtoco^®^ was found to have no increased safety risk in comparison to traditional forms of diazepam [63]. This new intranasal form of diazepam will help improve the lives of patients suffering with epilepsy.

## Figures and Tables

**Table 1:** Recommended dosage for adults and pediatric patients six years of age and older.

Dose Based on Age and Weight			Administration	
6 to 11 Years of Age (0.3 mg/kg) Weight (kg)	12 Years of Age and Older (0.2 mg/kg) Weight (kg)	Dose (mg)	Number of Nasal Spray Devices	Number of Sprays
10 to 18	14 to 27	5	One 5 mg device	One spray in one nostril
19 to 37	28 to 50	10	One 10 mg device	One spray in one nostril
38 to 55	51 to 75	15	Two 7.5 mg devices	One spray in one nostril
56 to 74	76 and up	20	Two 10 mg devices	One spray in one nostril

**Table 2:** Clinical efficacy and safety.

Author (Year)	Groups Studied and Intervention	Results and Findings	Conclusions
V. D. Ivaturi et al., 2009 [66]	Four subjects were recruited for a crossover study of intranasal and intravenous diazepam and midazolam. Maximum plasma concentration and time to maximum plasma concentration were recorded. Treatment-emergent adverse events were also monitored and recorded.	There was a higher maximum plasma concentration and increased time to reach maximum concentration for intranasal diazepam compared to intranasal midazolam. Nasal administration of both drugs elicited considerable pain in subjects.	Intranasal administration of both diazepam and midazolam were absorbed quickly. Diazepam has a longer half-life than intranasal midazolam.
Agarwal et al., 2013 [64]	A randomized open-label, six-sequence, three-way crossover study examined the effects and efficacy of intranasal suspension, solution, and intravenous diazepam. Maximum plasma concentration, time to maximum plasma concentration, and treatment-emergent adverse events were recorded.	The systemic availability of the intranasal solution was 97% compared to 67% of the intranasal suspension. There was a peak plasma concentration of 270 ng/mL for the 10 mg intranasal solution. Peak plasma concentration of the drug was attained approximately 60 min after administration. The mean plasma concentration of the intranasal solution was more than 100 ng/mL 8 h after administration; 71% of participants experienced mild or moderate treatment-emergent adverse events, but they were self-limiting.	Intranasal diazepam is a socially acceptable formulation, and shows comparable pharmacokinetics to rectal gel formulations. The intranasal spray is quick, easy to use, and well-tolerated for the treatment of seizures.
V. Ivaturi et al., 2013 [64]	Twelve healthy patients were enrolled in a crossover that compared the bioavailability, safety, and efficacy of intranasal diazepam compared to diazepam rectal gel. Maximum plasma concentration and time to maximum plasma concentration were recorded. Treatment-emergent adverse events, such as tolerance and sedation, were also monitored through visual analog scales and recorded.	The mean maximum plasma concentration was comparable in the 10 mg nasal spray and 10 mg rectal gel, and higher in the 13.4 mg nasal spray formulations. The maximum concentration was in the range of 150–190 ng/mL, which fell short of the common literature reported values. The time to maximum plasma concentration was 0.75 for both drugs. Subjects reported mild to moderate discomfort. Increasing sedation occurred, and its maximum effects occurred at 2 h after dosing, but did not correlate with the increase in dose.	Intranasal administration of diazepam is safe and easier than the rectal administration of the drug.
Henney et al., 2014 [63]	A randomized phase one crossover study that compared the pharmacokinetics and tolerability of intranasal diazepam to diazepam rectal gel in 24 subjects. Subjects received 5 mg or 20 mg of intranasal spray or 20 mg of rectal gel. Treatment-associated adverse events were monitored. C-SSRS was used to evaluate suicidal ideation.	The bioavailability of the 20 mg rectal gel was comparable to the 20 mg intranasal spray, regardless of the leakages from the nasal spray. The maximum plasma concentration of the 20 mg nasal spray and 20 mg rectal gel was 378 ng/mL and 328 ng/mL, and achieved within 1 h and 1.5 h, respectively. All of the participants reported at least one TEAE, with lacrimation being the most common. Others included but were not limited to sneezing, dysgeusia, and rhinorrhea. Somnolence was greater with the rectal gel and increased with increased dose. No suicidal ideation was reported by any of the subjects.	Intranasal and rectal gel diazepam were well-tolerated, with no case of a severe adverse event. Intranasal formulation presents with a socially acceptable and easy to use form of diazepam to use in seizure rescue.
Sperling et al., 2014 [67]	Thirty patients were enrolled, with 10 treated during a tonic-clonic seizure episode, seven treated within 5 min of seizure cessation, and 13 dosed 5 min or more after a seizure. Maximum plasma concentration, time to maximum plasma concentration, and concentration over a 12 h period were recorded. Treatment-emergent adverse events were also monitored	The mean time to reach maximum plasma concentration was 45 min, and the maximum plasma concentration and concentration over 12 h was comparable in the three groups; 65% of the subjects experienced no seizure within the 12 h observation period, while 35% experienced seizure afterward, and 90% of subjects experienced at least one adverse event, with headache being the most common.	The effective therapeutic concentration of diazepam can be delivered through the nasal spray in the ictal state or post-ictal state without significant adverse events locally or systemically.
Inokuchi et al., 2015 [68]	A retrospective cohort study examined the use of intranasal vs. intravenous diazepam in previously diagnosed stroke patients in status epilepticus. Data from 19 patients were involved in the study. The time to cessation of seizure after arrival and after dosing was recorded. Adverse events were also monitored.	The administration of diazepam intranasally was nine times faster than intravenous administration. This difference was statistically significant. The cessation of active seizure was 3 min for intranasal administration and 0.5 min in intravenous administration, but this difference was not statistically significant. There was no severe adverse event reported in this study.	Intranasal diazepam is safe, quick, and easy to use in a patient in status epilepticus. This would make the administration of diazepam outside of the hospital setting easier.
R. Edward Hogan et al., 2020 [46]	Forty-eight healthy subjects were enrolled in a randomized crossover study that compared the bioavailability and safety of diazepam rectal gel to intranasal spray. Oral diazepam was included as a control in the study. The time to maximum plasma concentration, peak plasma concentration, and variability were recorded. Treatment-emergent adverse events were also monitored.	The absorption of diazepam for both formulations was rapid, and the time to reach the maximum plasma concentration was comparable. There was a difference in variability, with the rectal gel showing greater variability compared to the nasal spray. Subjects with greater weight showed higher variability compared to those with lesser weight. There were 131 cases of TEAEs, with every subject experiencing TEAEs at least once. The TEAEs were mild to moderate, and no case of serious TEAE was reported.	Diazepam nasal spray shows great bioavailability, good tolerance, and is safe for the control of cluster seizures.
Robert Edward Hogan et al., 2020 [47]	Fifty-seven patients were given 10 mg, 15 mg, or 20 mg doses of diazepam once in either ictal/pericital or interictal periods. The patients’ age ranged from 6–65 years. Treatment-emergent adverse events from this study were recorded.	The mean plasma concentration of diazepam after administration was similar during ictal/perictal (164 ng/mL) and interictal (189 ng/mL) periods. Seventeen patients reported TEAEs. One patient had a serious adverse effect that was not related to the treatment. There was no difference in respiration, sedation, or pain from baseline.	Diazepam nasal spray is safely administered during status epilepticus episodes, with comparable effects in the time of administration. Therefore, it is safe to be administered at any time.

## Data Availability

Not applicable.
